# Regulation of T Helper 17 by Bacteria: An Approach for the Treatment of Hepatocellular Carcinoma

**DOI:** 10.1155/2012/439024

**Published:** 2012-12-17

**Authors:** Cecilia Ying Ju Sung, Nikki Pui-yue Lee, Hani El-Nezami

**Affiliations:** ^1^School of Biological Sciences, Faculty of Sciences, Kadoorie Biological Sciences Building, The University of Hong Kong, Pokfulam, Hong Kong; ^2^Department of Surgery, LKS Faculty of Medicine, The University of Hong Kong, Pokfulam, Hong Kong

## Abstract

T helper 17 (T_H_17) is a novel subset of T helper cells that has recently been identified in the hepatocellular carcinoma (HCC) tumor environment. Its presence seems to be linked with HCC progression, possibly via facilitating angiogenesis. The origin of tumor-associated T_H_17 may be related to the gut, in which the differentiation of T cells, especially T_H_17 cells, is affected by microbiota. As T_H_17 may appear to be a new therapeutic target against tumor-promoting inflammation, strategies such as using probiotics to polarize the response away from T_H_17 may be beneficial to slow down tumor progression. This paper will attempt to discuss the potential linkage between HCC progression, T_H_17, and gut microbiota and the possible therapeutic implications of probiotics to modulate T_H_17-mediated response for tumor growth.

## 1. Introduction 

Hepatocellular carcinoma (HCC) is the fifth most common cancer worldwide and is characterized by poor prognosis [1]. Tumor progression has now been recognized as the product of crosstalk between cancer cells and stromal cells, including immune cells [2]. Immune status appears to be different in distinct sites of the tumor [3]. The intratumoral region is generally in an immunosuppressive state [3] it contains dysfunctional antigen presenting cells, altered proportion of effector to regulatory T cells, and an abundance of immunosuppressive molecules, forming a network to facilitate immune evasion [4]. In contrast, the peritumoral stroma is highly infiltrated with various immune cells that actively secrete high concentrations of inflammatory cytokines for enhancing cell growth, angiogenesis, and tissue remodelling [3]. Hence, inflammatory response has been suggested to be rerouted in a tumor-promoting direction. Recently, T_H_17 cells have come into research focus as they have been identified in a number of tumors including HCC. T_H_17, and its effector molecules interleukin-17 (IL-17) and IL-22, are potent inducers of tissue inflammation and have been associated with a number of inflammatory and autoimmune diseases [5, 6]. The role of T_H_17 is paradoxical, but now there is accumulating evidence to illustrate that T_H_17 has tumor promoting effects in some cancer such as HCC. Though the origin of tumor associated T_H_17 cells is not completely understood, it is possible that they are recruited from the periphery [7]. The gut is the natural site of T_H_17 generation and it has recently been found that microbes can affect T cell differentiation via regulating dendritic cells. Thus, there appears to be a complex relationship between HCC progression, T_H_17 and gut microbiota. In this paper, the potential linkage between these three factors and the possible therapeutic implications of probiotics to modulate T_H_17-mediated response for tumor growth will be discussed.

## 2. Relationship between IL-17-Producing Cells and HCC Progression

IL-17 is a proinflammatory cytokine produced primarily by a novel subset of CD4+T cells known as T_H_17. In addition to T_H_ cells, this cytokine can also be secreted by CD8+T cells, *γδ* T cells, lymphoid tissue inducer (LTi) cells, natural killer (NK) cells, and granulocytes [8]. At present, the IL-17-producing cells in human HCC tissue are found to be from the adaptive arm of immunity. A majority of them were identified to be T_H_17, though a substantial amount of IL-17+CD8+T cells can also be found in tumor. In addition to IL-17, these cells may also secrete IL-22, which was recently found to be related to HCC as well, though its production is not limited to T cells [9–11].

The role of T_H_17 cells in tumor immunity has been controversial. However, several lines of evidence suggested that these cells play a protumor role in HCC. Increased levels of T_H_17 cells were found in tumor tissue [12] and in peripheral blood [13] of HCC patients, and their level is correlated with unfavorable disease outcomes [7, 12, 14]. Similar results have also been observed in animal models, whereby limiting tumor T_H_17 expansion reduced the growth of transplanted liver tumor in rodents [7]. 

Many functions of IL-17 in the tumor microenvironment contribute to tumor progression. Apart from a minor direct effect on the proliferation and survival of cancer cells in other systems [15], and the recent report on its role in immune evasion via mediating B7-H1 expression on monocytes to suppress cytotoxic T cell activity [13], the major protumor role of IL-17 in inflammation-associated cancer relies on fostering angiogenesis. Indeed, both animal and human HCC tissues revealed that their levels were positively correlated with microvessel density and that these cells were observed to be enriched predominately at the invading edge of tumor tissue, the site where angiogenesis is most active [12]. The proangiogenic effect of IL-17 could be linked to its interaction with various stromal cells such as fibroblasts, keratinocytes, epithelial and endothelial cells. IL-17 leads to the induction of IL-6, IL-8, prostaglandin (PG) E_1_, and PGE_2_ as well as enhancement of intercellular adhesion molecule-1 expression [16–19]. IL-17 also participates in mobilization and recruitment of neutrophils [20] as well as inducing the secretion of tumor necrosis factor-*α* (TNF-*α*) and IL-1*β* from macrophages [21]. The collective effect results in the release of an array of proangiogenic cytokines including vascular endothelial growth factor (VEGF), hepatocyte growth factor and keratinocyte-derived chemokine in the tumor microenvironment [22]. Thus, IL-17 inevitably shifts the local biologic balance toward a predominance of angiogenic factors to enhance the net angiogenic activity. Owing to the highly vascular nature of HCC, IL-17+ cells may play an important role in progression of this type of tumor.

In addition to IL-17, it is conceivable that T_H_17 cell promote HCC via IL-22 production as well. Though there were only a few reports on the role of IL-22 in tumors, the literature generally supported the tumor-promoting function of this cytokine. A recent report from Jiang et al. [10] illustrated the excessive expression of IL-22 in HCC microenvironment and its expression appears to be related to advanced cancer stages. Conversely, knockdown of IL-22 inhibited tumor progression in a xenograft model of other systems [23]. The tumor promoting effect of IL-22 is believed to be mediated by signal transducer and activator of transcription factor 3 (STAT3), an oncogenic transcription factor constitutively activated in various malignancies [24]. In human liver cancer cells, IL-22-induced STAT3 activation promoted at least three hallmarks of cancer (proliferation, survival and angiogenesis) via the upregulation of a variety of mitogenic (cyclin D1, c-myc, and Rb2), anti-apoptotic (Bcl-2 and Bcl-xL) [10, 25], and angiogenic (VEGF) [10] genes. IL-22 was also shown to have immunosuppressive functions in other cancer [26], though it was not well studied in HCC so far.

Since T_H_17 cells may act to promote HCC pathogenesis via production of IL-17 and IL-22; if we can modulate the T_H_17 status in the body, it may be possible to affect tumor progression. In order to do this, we first need to know the origin of tumor-associated T_H_17 cells.

## 3. Potential Source of Tumor-Associated T_H_-17 Cells

Tumor-associated T_H_17 cells may either be induced in the tumor microenvironment and/or recruited from distal sites.

*In situ* induction occurs when memory T cells enter a site of inflammation and encounter activated antigen-presenting cells (APC). Though it has been suggested that tumor-associated-macrophage (TAM) may be responsible for T_H_17 development because it outnumbers dendritic cells (DC), the most efficient APC, in the tumor environment, it appears that tumor-activated monocytes, but not TAM may play a dominant role in T_H_17 expansion in the context of HCC. Kuang et al. [7] have shown that monocytes could be activated by liver cancer cells to secrete several cytokines including IL-1*β*, IL-6, IL-23, thereby creating a proinflammatory cytokine milieu that facilitates the *in vitro* expansion of memory T cells. While studies in other systems have also demonstrated a critical role of transforming growth factor-*β* (TGF-*β*) in the development of human T_H_17 [27, 28], Kuang and his colleagues failed to find a correlation between the expression of this cytokine and T_H_17 cell density, implicating that the role of TGF-*β* in expanding T_H_17 cells remains to be elucidated in the local tumor environment of HCC. It is interesting to note that T_H_17 cells generated in this cytokine milieu can also produce interferon-*γ* (IFN-*γ*), which is the signature cytokine of T_H_1 cells. Thus there are two subsets of T_H_ cells in HCC tissue: T_H_17 (IL-17+ IFN-*γ*−) and T_H_17/T_H_1 (IL-17+ IFN-*γ*+). How T_H_17/T_H_1 cells are generated is not yet known but it is possible that the tumor environment induces this phenotype, as most of the circulating T_H_17 cells in HCC patients did not express IFN-*γ*. IFN-*γ* from T_H_17/T_H_1 is suggested to promote further recruitment of T_H_17 by inducing CCL20 expression. CCL20 is the ligand for CCR6, which is a receptor highly expressed in the majority of T_H_17 cells [7, 29]. A positive feedback cycle may be formed in the tissue environment: CCR6+ memory T_H_17 is homed to tumor site by high levels of CCL20 [12]. It is then converted to T_H_17/T_H_1, which releases IFN-*γ* to recruit more CCR6+ memory T_H_17 from the periphery pool by the virtue of CCL20. 

**Figure 1:**
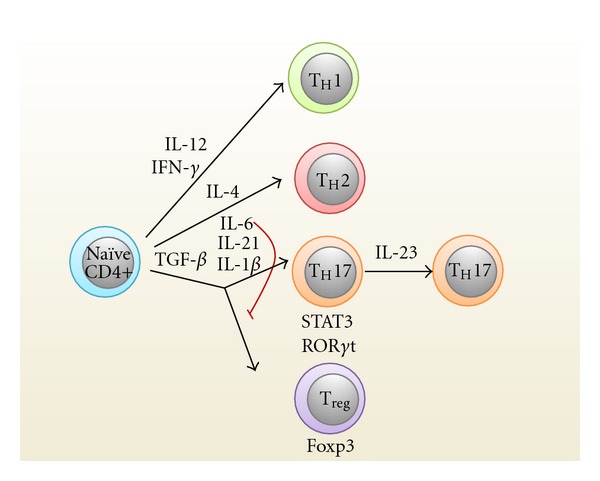
Polarization of T helper (T_H_) cell subsets. Naïve CD4+ T cells develop into different lineages (T_H_1, T_H_2, T_H_17, and T_reg_) in response to cytokine cues produced from antigen presenting cells. IL: interleukin; interferon *γ*: IFN-*γ*; FOXP3: forkhead box P3; ROR*γ*t: retinoic acid-related orphan receptor *γ*t; STAT: signal transducer and activator of transcription; *β*: TGF-*β* transforming growth factor.

So where would the potential source for T_H_17 cells be? T_H_17 cells are preferentially enriched in the intestinal lamina propria of ileum and colon, while very low frequencies of these cells are present in extraintestinal sites at a steady state [30–32]. Polarization of naïve T cells is influenced by diverse signals produced by APC to develop into distinct effector (T_H_1, T_H_2, and T_H_17) or regulatory (T_reg_) lineages (Figure 1). It is now recognized that IL-12 and IFN-*γ* are needed for T_H_1 induction, whereas T_H_2 differentiation requires IL-4, and T_reg_ requires TGF-*β* [33]. For T_H_17 differentiation, *in vitro* studies show that it requires multiple cytokines including TGF-*β*, IL-6, IL-21, IL-23, and IL-1*β* [8, 34–38]. TGF-*β* induces the expression of the retinoic acid-related orphan receptor ROR*γ*t, which is the master transcription factor for the T_H_17 effector cell lineage [30]. However, TGF-*β* alone is unable to initiate T_H_17 differentiation, as this cytokine also induces forkhead box p3 (Foxp3), which is a transcription factor essential for the differentiation of T_regs_. Foxp3 would bind to ROR*γ*t and thereby inhibit ROR*γ*t-directed IL-17 expression [39], and hence excess TGF-*β* can inhibit expression and function of ROR*γ*t. In the presence of proinflammatory cytokines such as IL-6 or IL-21, this inhibitory effect would be relived, as these cytokines activate STAT3 and suppress the expression of Foxp3. As a result, the relative levels of ROR*γ*t are increased and T_H_17 cell differentiation is promoted. Once T_H_17 cells have developed, IL-23 is needed for stabilization and further expansion of these cells, as illustrated by studies with IL-23 receptor-deficient mice [40].

## 4. The Role of Microbiota in T_H_17 Immunity

Since IL-23 is required for developing productive and sustained T_H_17 responses, the signals that induce the production of this cytokine might be critical in determining whether T_H_17 cells dominated T cell response. Given that IL-23 is mainly produced by innate immune cells, including DCs and macrophages in the gut, it is not surprising to find that signals from commensal bacteria is necessary for induction of T_H_17 cells. This notion is supported by observation where T_H_17 cells were absent in the sterile gut of newborn mice but steadily increased from birth to post-weaning as symbiotic bacteria gradually colonized the intestine [41]. More importantly, it is not general bacterial colonization but the composition of bacteria that influence the makeup of the lamina propria T lymphocyte subsets. Ivanov et al. [32] has found that mice purchased from different vendors had shown marked differences in the number of T_H_17 cells in the gut. By sequencing microbiota of these animals, it was found that T_H_17 cell responses appear to be induced by specific classes of bacteria known as segmented filamentous bacteria (SFB), a Gram-positive bacteria belonging to the *Firmicutes* phylum and most closely related to the  *Clostridium* genus [42]. Prominent T_H_17 responses may also be seen upon infection of pathogenic  *Mycobacterium* [43], *Klebsiella* [44], and *Citrobacter* [36] or upon colonization with a complex microbial community [45]. Since multiple chronic liver diseases including HCC are often associated with intestinal dysbiosis and reduced species diversity [46–48], it seems that gut-derived microbial signals and intestinal immune network may be a factor to potentially reprogram systemic immune response towards a tumor-promoting direction.

**Figure 2:**
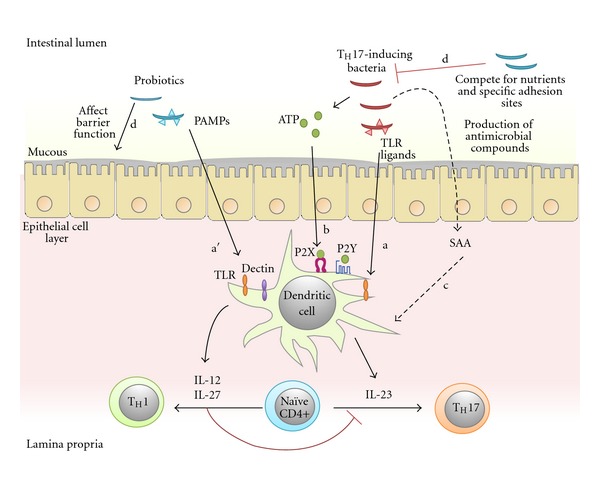
A simplified diagram showing the possible mechanisms of intestinal bacteria in influencing the polarization of T_H_17 cells in the lamina propria. Activation of dendritic cells by intestinal microbes results in secretion of proinflammatory cytokines such as IL-12, L-23, IL-27. T_H_17-inducing bacteria may promote T_H_17 immunity via IL-23 induction, which may involve signaling mediated by the TLR ligands (a), extracellular ATP (b), and SAA (c). Meanwhile, some probiotic strains may skew immunity away from T_H_17 via IL-12 and IL-27 induction as a result of activating TLR and dectin receptors (a′). These cytokines can inhibit T_H_17 development while facilitate T_H_1 differentiation. Probiotic may also work by controlling the growth and colonization of T_H_17-inducing bacteria (d). IL: interleukin; P2X: ionotropic receptors; P2Y: metabotropic receptors; PAMPs: pathogen-associated molecular patterns; SAA: serum amyloid A; TLR: toll-like receptor.

The mechanisms of how intestinal bacteria prime DC for T_H_17 development is not yet fully understood, but it is likely to involve several microbial-derived molecules such as the toll-like receptor (TLR) ligands, adenosine 5′-triphosphate (ATP), and serum amyloid A (SAA) that result in IL-23 production. TLRs are a class of pattern-recognition receptors (PRRs) that play a key role in the innate immune system for recognizing various microbes and/or its products, collectively known as pathogen-associated molecular patterns (PAMPs) [49]. The nature of cytokines secreted by DC is dependent on PAMPs that DC encountered in the peripheral tissues during its immature phase. While stimulation of TLR4 by LPS gives both IL-23 and IL-12, stimulation of TLR2 by peptidoglycan generally induces large amounts of IL-23 from DC, though the quantity may vary depending on the structure of peptidoglycan [50]. TLR9 and TLR5 signaling may also be necessary, as illustrated by *in vivo* [51] and *in vitro* [52] studies, respectively. Apart from PAMPs, the binding of extracellular ATP could also elicit the release of IL-23 in addition to other IL-17 inducing proinflammatory cytokines such as IL-6 and IL-1*β* from DC [31, 53, 54] as a result of activating the membrane ion channel and G protein receptors such as ionotropic and metabotropic purinergic receptors [55, 56]. The importance of the ATP signaling pathway was demonstrated *in vitro*, whereby the differentiation program of T_H_17 became severely inhibited upon addition of ATP degrading enzyme apyrase [31]. Commensal bacteria have been shown to generate copious amount of extracellular ATP [57] and thereby important in IL-23 production. Transient production of IL-23 by lamina propria DCs can also be induce by SAA, an acute phase protein found to be upregulated in the ileum by T_H_17 inducing bacteria. However, the signaling pathways induced by SAA are currently unknown [58]. Collectively, these findings illustrate that microbiota played an important role in sustaining T_H_17 responses via IL-23 induction, which may involve signaling mediated by the TLR ligand, ATP, and SAA. This mechanism establishes a T_H_17 cell-inducing cytokine environment (Figure 2).

## 5. Modulation of Extraintestinal T_H_17 Response by Commensal Bacteria

Notably, the influence of commensal bacteria on the balance of T cell subsets is not only confined to the gut, but can also be extended to extraintestinal sites. This notion may be supported by several studies of autoimmune disease whose development is T_H_17-dependent. It was found that introduction of SFBs into the sterile gut of healthy mice was able to induce arthritis and encephalomyelitis [59, 60]. The aggravation of disease appeared to be the consequence of an increase in the number of T_H_17 cells that traffic out of the gut to the extraintestinal site, as Lee et al. [59] has revealed an increase in T_H_17 cell responses in spinal cords of SFB monocolonized mice in a model of encephalomyelitis. These findings may have significant implications for regulating systemic pathogenic T_H_17 by modulating the composition of intestinal bacteria, and probiotics have been suggested to exhibit this potential.

## 6. Potential Immunomodulatory Capacity of Probiotics 

Probiotics are live microorganisms that confer health benefit to host when administered in adequate amounts [61]. The established probiotics are generally *Lactobacillus* and  *Bifidobacterium* species, though *Lactococcus*, *Enterococcus*, and *Streptococcus* species, as well as some nonpathogenic strains of *Escherichia coli* that can also be found [62]. Administration of *Lactobacillus* and *Bifidobacterium* species orally has been shown to protect against the development of various T_H_17-mediated diseases [63–72], possibly via reprogramming T_H_ cell response or by controlling growth of T_H_17-inducing bacteria. Due to strain or species-specific molecular characteristics of probiotic bacteria, the immunomodulatory effect exhibited may depend strongly on the choice of the probiotic strain.

Probiotic may prime DC for the development of other T_H_ subsets. For example, activation of TLR or dectin receptors would trigger DC to produce IL-12p70, IL-23, or IL-27, which are important in skewing T_H_1 or T_H_17 immunity. While IL-23 sustains T_H_17 response, IL-12 and IL-27 drive T_H_1 differentiation by activating STAT1 and inducing the expression of T box transcription factor (T-bet), the key transcriptional regulator of T_H_1 cells [5]. IL-27 and IFN-*γ* derived from T_H_1 cells could downregulate ROR*γ*t in a STAT1-dependent manner and thereby dampen development of T_H_17 cells, whereas IL-17 from T_H_17 did not similarly suppress T_H_1 polarization [73]. IL-12 and IFN-*γ* would further increase T-bet expression in DC to drive T_H_1 differentiation [74]. Hence, probiotic strain that favors IL-12 and IL-27 is likely to skew immune response away from T_H_17 to T_H_1 (Figure 2). Several strains of *Lactobacillus *(e.g., *L. acidophilus* [75], *L. gasseri* [72], and *L. rhamnosus* [76]) and *Bifidobacterium* (e.g., *B. lactis*, *B. breve* and *B. bifidum* [77, 78]) may possess this capacity. T_H_1 cytokines such as IL-12 and IFN-*γ* are known to have potent antitumor immunity as they could activate cytotoxic T cells and NK cells to kill cancer cells. Deficiency of T-bet in DC leads to exaggerated TNF production and contributes to creating a chronic inflammatory state that modulates the composition of microbiota and eventually leads to cancer development [79, 80]. Therefore, promoting T_H_1 differentiation by probiotics may possibly shift pathogenic T_H_17 inflammation to antitumor T_H_1 response.

Apart from regulating T cell polarization, probiotic may also work by controlling the growth of T_H_17-inducing bacteria such as SFB. Indeed, administration of  *L. plantarum* almost completely depleted the SFB present in the ileum [81]. Although the underlying mechanisms have not yet been investigated in that study, it is tempting to speculate that probiotic may adversely affect SFB colonization and survival by competing for the use of nutrients and other external metabolites. In accordance to the highly reduced genome, SFBs lack a number of enzymes for basic metabolic pathways that are important for growth and survival, including biosynthesis of amino acids and cofactors. To compensate for these auxotrophies, SFB expressed a large array of transporters to acquire sugars, many cofactors, and nearly all amino acids and from the environment [82]. *Lactobacillus* may serve as a competitor for the uptake of amino acids, as genome sequencing has revealed a considerable degree of auxotrophy for amino acids in these bacteria. There may also be competition for the internalization and utilization of sugar, as *Bifidobacterium* and *Lactobacillus* situated in intestinal niche generally encode a large capacity for carbohydrate transport and metabolism [83–87]. *Bifidobacterium* have excellent carbohydrate sequestering capacity as they use a “docking station” to capture carbohydrate to their cell surface to avoid losing the molecules to nearby competitors [88–90]. In addition to amino acids and sugar utilization, the metabolism of some *Lactobacillus* strains, including *L. paracasei*  or  *L. rhamnosus*, could also lead to major changes to levels of a number of metabolites, including methylamines and short-chain fatty acids [91, 92]. Together, the metabolisms and activities of these commensal bacteria may create an unfavorable environment for functionality of SFB *in vivo*. Other strategies for probiotics to limit pathogenic bacterial growth may include production of antimicrobial compounds, competition for specific adhesion sites and maintaining intestinal tight junction [81], but these mechanisms will not be discussed here in detail. All these mechanisms may directly or indirectly change the composition and diversity of the intestinal microbiota and modulate DC-mediated immunity as mentioned above.

All in all, commensal bacteria of *Bifidobacterium* and *Lactobacillus* genera are associated with balancing T_H_ response locally and systemically. Hence, establishing a balanced microbiota in favor of these protective probiotic bacteria may be a good strategy to maintain immune homeostasis via DC priming and that may possibly modulate tumorigenic proinflammatory milieu at sites distant from the gut.

## 7. Conclusion 

In conclusion, T_H_17 has recently been found in HCC tumor and its presence has been linked to disease progression, possibly involving angiogenesis. Gut T_H_17 seems to be a potential source for tumor-associated T_H_17, where it could be homed to the tumor environment via CCR6/CCL20 axis and expand locally. Commensal bacteria are necessary for development of gut T_H_17 by IL-23 induction in DC. Probiotics may affect cytokine profile of DC by activating different PRRs and controlling the growth of some potent T_H_17 inducers such as SFB. This potential linkage of HCC environment, T_H_17 cells, and microbiota may implicate for novel targets for therapeutic intervention in HCC progression.
